# Measuring Complexity in Manufacturing: Integrating Entropic Methods, Programming and Simulation

**DOI:** 10.3390/e27010050

**Published:** 2025-01-09

**Authors:** Germán Herrera-Vidal, Jairo R. Coronado-Hernández, Ivan Derpich-Contreras, Breezy P. Martínez Paredes, Gustavo Gatica

**Affiliations:** 1 Industrial Engineering School, Universidad del Sinú, Cartagena 130001, Colombia; 2 Department of Productivity and Innovation, Universidad de la Costa, Barranquilla 080016, Colombia; 3 Faculty of Engineering, Universidad de Santiago de Chile, Santiago 8370003, Chile; 4 Faculty of Engineering, Universidad Nacional Mayor de San Marcos, Lima 15081, Peru; bmartinezp1@unmsm.edu.pe; 5 Faculty of Engineering, Universidad Andres Bello, Santiago 8370146, Chile; ggatica@unab.cl

**Keywords:** complexity, methodology, entropic, measurement, manufacturing systems

## Abstract

This research addresses complexity in manufacturing systems from an entropic perspective for production improvement. The main objective is to develop and validate a methodology that develops an entropic metric of complexity in an integral way in production environments, through simulation and programming techniques. The methodological proposal is composed of six stages: (i) Case study, (ii) Hypothesis formulation, (iii) Discrete event simulation, (iv) Measurement of entropic complexity by applying Shannon’s information theory, (v) Entropy analysis, and (vi) Statistical analysis by ANOVA. The results confirm that factors such as production sequence and product volume significantly influence the structural complexity of the workstations, with station A being less complex (0.4154 to 0.9913 bits) compared to stations B and C, which reached up to 2.2084 bits. This analysis has shown that optimizing production scheduling can reduce bottlenecks and improve system efficiency. Furthermore, the developed methodology, validated in a case study of the metalworking sector, provides a quantitative framework that combines discrete event simulation and robust statistical analysis, offering an effective tool to anticipate and manage complexity in production. In synthesis, this research presents an innovative methodology to measure static and dynamic complexity in manufacturing systems, with practical application to improve efficiency and competitiveness in the industrial sector.

## 1. Introduction

Manufacturing systems are complex structures that transform raw materials into finished products through the integration of technology, machinery, labor, and production methods. Efficient management of these systems requires continuous monitoring due to the inherent randomness of the variables that affect them, which makes it essential to develop accurate and useful metrics to evaluate key aspects such as production times, productivity, efficiency, product quality, inventory, and operating costs [1]. These metrics not only facilitate informed decision-making but also help to identify critical areas for improvement and maintenance of operational control [2]. In this context, the concept of complexity has emerged as a key approach to assessing the performance of manufacturing systems. According to the Oxford Learner’s Dictionary [3], complexity is defined as the state of being composed of multiple interconnected and difficult-to-understand elements. A complex system is characterized by interactions related to size, quantity, asymmetries, and randomness [4], and also incorporates the requirement for information as a key factor [5]. In manufacturing systems, this is reflected in the high variety, diversity, and uncertainty of their processes [6,7,8]. As firms expand their capacity to cope with demand volatility, their operations tend to become more complex, facing problems such as machine breakdowns, material defects, and labor absenteeism [9]. Recent studies claim that complexity accounts for up to 25% of total costs in manufacturing firms due to the process and associated product characteristics [2]. This reinforces the need to develop specific methods and techniques to measure and manage complexity, which has become an essential tool for operational improvement and business restructuring [10].

The literature distinguishes different approaches and methods for measuring complexity, among which are nonlinear dynamics, information theory (entropic), hybrid methods, quantitative indexes, and other approaches. Similarly, different types of models are classified from a conceptual, theoretical, and mathematical perspective [11]. With greater inclination towards objective, analytical methods, and mathematical models, based on optimization techniques and process simulation [12]. Table 1 synthesizes in a structured manner the main gaps and challenges identified in the current literature on advanced manufacturing systems. The gaps correspond to limitations in the existing knowledge, such as the limited application to real industrial environments, the lack of validations in diverse systems and the predominance of theoretical formulations. These gaps highlight critical areas where further research is required to close the gap between theory and practice. On the other hand, the challenges represent conceptual and practical obstacles that hinder the implementation of solutions, such as expansion to larger and more diverse systems, empirical validation in complex industrial environments, and integration of real-time human-machine interaction. Separating gaps and challenges makes it possible to identify, on the one hand, gaps that need to be addressed by future research and, on the other hand, practical obstacles whose overcoming is necessary to move towards applicability and scalability of systems in real environments. This differentiation provides a clear basis for setting priorities in both academic research and technological development.

The review of studies on complexity measurement in manufacturing systems reveals limited application in real industrial environments and lack of empirical validation in a variety of systems, especially those with theoretical formulations. In addition, various approaches focus on specific systems or limited flows, which restricts their generalizability. In terms of challenges, the need to extend the applicability of these methods to dynamic systems, improve measurement accuracy and empirically validate the techniques in complex industrial environments are highlighted.

This paper represents a significant advance in a consolidated line of research that the authors have been developing in the field of complexity in manufacturing systems, ranging from comprehensive literature reviews (Complexity in Manufacturing Systems: A Literature Review, 2021) [6], to the critical evaluation of traditional models and metrics (Measuring Manufacturing System Complexity: A Literature Review, 2022) [11] and statistical analysis applied to complex production configurations (Statistical Analysis of Manufacturing System Complexity, 2022) [8]. Building on this foundation, this study innovatively integrates Shannon information theory with discrete event simulation techniques, proposing a comprehensive methodological framework that allows for a simultaneous analysis of static and dynamic complexity, overcoming the limitations of traditional approaches focused exclusively on static perspectives (Modeling and Statistical Analysis of Complexity in Manufacturing Systems Under Flow Shop and Hybrid Environments, 2022) [26]. In addition, comparative analysis using conditional measures and entropies is advanced, as recently explored in innovative metrics (Measuring Complexity in Manufacturing Systems: A New Metric in Flow Shop (FS) and Job Shop (JS) Environments, 2023) [27]. Validated in a practical environment in the metalworking sector, this approach not only demonstrates its applicability to complex industrial scenarios but also offers a solution adaptable to other production sectors. In synthesis, this work redefines the understanding and management of complexity in manufacturing through robust methodological tools that optimize interpretation and operational decision-making, positioning itself as an original and transformative contribution to the field.

The main contributions of this work are the following:

(i) Integrated methodology for measuring complexity: This work presents an innovative approach that combines the simulation of discrete events with the calculation of complexity using Shannon entropy. This allows simultaneous and more complete analysis of both static and dynamic complexity in manufacturing systems, overcoming the limitations of traditional methods that focus exclusively on a single type of complexity.
(ii) Advances in dynamic and comparative analysis: Unlike conventional approaches, this study introduces a dynamic analysis of structural and functional complexity, using comparative and conditional measures of entropy. This allows for optimized interpretation and decision-making, providing a deeper and more robust perspective on the behavior of production systems under varying conditions.
(iii) Empirical validation and practical relevance: The developed methodology has been applied and validated in a practical case within the metal-mechanical sector, demonstrating its applicability for the evaluation of real manufacturing systems. This validation highlights the usefulness of the approach as a key tool to improve efficiency, reduce bottlenecks, and guide strategic decisions in industrial management.

The remainder of the paper is organized as follows. Section 2 develops a literature review. Section 3 describes and proposes the materials and methods. Section 4 presents the results. Section 5 presents the discussion of the research results. Section 6 presents the conclusions and some possible future studies.

## 2. Literature Review

### 2.1. Definition and Foundations of Complexity

One of the earliest definitions of complexity comes from the law of variety of requirements, proposed by [28], which states that only complexity can absorb complexity. This idea emphasizes that as complexity increases in a system, its control must also increase. In the context of manufacturing systems, where the goal is to add value through efficient processes [29], multiple resources such as infrastructure, materials, machines, and people interact with one another [30]. From an external perspective, this complexity also includes factors such as customers, markets, and suppliers [31]. To ensure equilibrium, Ulrich and Probst [32] stress the need for stability, heterogeneity, and diversity in the elements of the system.

### 2.2. Complexity as a Challenge in Manufacturing Systems

Complexity in manufacturing systems is multifaceted, arising from the interaction between elements and technologies that make optimization difficult [33]. Factors such as system structure, product variety, and process interconnectedness have been identified as key determinants [34]. Emerging technologies add additional levels of complexity, especially in dynamic environments [35]. Recent studies highlight the direct influence of these interactions on system performance [36], making evident the need for systematic approaches to their management [37].

### 2.3. Classification of Complexity in Manufacturing Systems

Complexity can be classified according to its origin (internal or external) [38] or its behavior over time (static and dynamic) [39]. Static complexity analyses the structure of the system at a specific point in time [40], while dynamic complexity assesses its evolution and behavior in the face of change [41]. Addressing both dimensions is essential for managing variability and adaptability in modern industry [42].

### 2.4. Entropy as a Key Metric for Measuring Complexity

Information theory has established entropy as a central metric for assessing complexity in manufacturing systems. Studies such as those by [43,44] have applied entropy to quantify uncertainty and concentrations in production processes. Its use has been extended to the analysis of economic systems [45] and, more recently, to the assessment of dynamic complexity in industrial processes [46]. However, there are gaps in the practical integration of these metrics in operational management.

### 2.5. Simulation as a Tool for Complexity Management

Simulation has emerged as a critical tool for managing complexity in manufacturing systems, allowing interactions to be modelled and processes to be optimized [26]. Recent studies have highlighted its effectiveness in addressing both static and dynamic complexity [47], facilitating real-time data integration and adaptive strategy design [48]. Advanced simulation tools, combined with technologies such as Big Data, are emerging as the future of industrial complexity management [1,42].

The literature frames a number of works related to the use of simulation as a key tool to address this challenge, recent research contributions developed by [49] highlight the role of simulation in measuring operational complexity, especially in the interrelationship between suppliers and customers, while ref. [35] underlines how information technology, together with simulation, can facilitate the management of complexity in manufacturing processes. Ref. [26] presents a model that integrates simulation to evaluate both static and dynamic complexity, showing its application in different manufacturing scenarios. Ref. [47] performs a systematic review of complexity management methodologies, highlighting simulation as a central tool to optimize processes, a trend also supported by [36], which investigates the relationship between complexity and performance using simulation to model production scenarios. Ref. [41] proposes a framework that includes simulation as an essential component for assessing complexity in manufacturing, while ref. [50] identifies this methodology as key to addressing the challenges associated with complexity in industry. Ref. [26] analyzes the effectiveness of simulation in different contexts, arguing that its application may vary according to the complexity of the system and the specific characteristics of the production. Finally, refs. [12,27] offer a comprehensive analysis that highlights simulation as an indispensable tool for complexity management in manufacturing systems. In synthesis, simulation emerges as the most prominent tool for managing dynamic complexity in manufacturing systems, allowing modeling and optimizing processes in various scenarios. Current trends point to a greater integration of simulation technologies with real-time information to improve efficiency and adaptability in production.

The concept of complexity in manufacturing systems is defined as the dynamic interaction between components, processes, and environments, classified into static and dynamic complexity. Static complexity evaluates the structure and relationships at an instant, while dynamic complexity examines their evolution over time. Methods such as Shannon entropy are essential to quantify these complexities, facilitating the identification of inefficiencies and areas for improvement. In addition, the use of information and simulation technologies becomes crucial to manage this complexity, allowing agile adaptation to changes in demand and optimizing decision-making. The research focuses on developing integrated frameworks that address these dimensions, seeking to improve competitiveness and efficiency in manufacturing environments.

## 3. Materials and Methods

**Figure 1:**
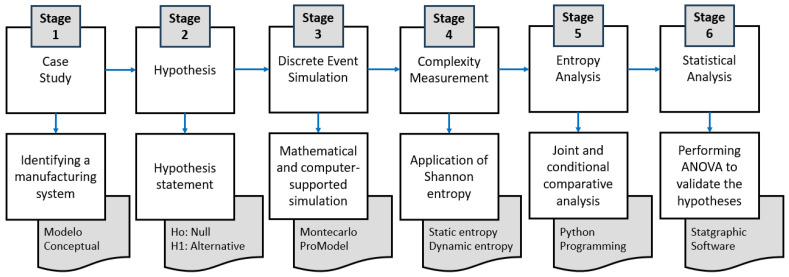
Methodological proposal for this research.

The methodological proposal of this research is structured in six stages that allow measuring and analyzing the complexity and behavior of manufacturing systems by means of discrete event simulation. Stage (i) begins with a case study, in which a specific manufacturing system is identified and a conceptual model is developed as the basis for the analysis; (ii) Hypothesis formulation, posing the null hypothesis (H_0_) and alternative hypothesis (H_1_); (iii) Discrete event simulation, using tools such as Monte Carlo and ProModel 10.0 to observe the behavior of the system; (iv) Entropic complexity measurement applying Shannon’s information theory, both for the static and dynamic complexity of the system; (v) Entropy analysis, using Python 3.11 programming that allows a joint and conditional comparative analysis; and (vi) Statistical Analysis using ANOVA to validate the formulated hypotheses, using specialized software such as Statgraphics 19 (see Figure 1).

### 3.1. Case Study

In order to achieve the objective based on the measurement of complexity in manufacturing systems, based on simulation, a practical study scenario is proposed, consisting of three (3) workstations, relating different operations, specific activities, processes, and different products. Table 2 details the integral flow of the manufacturing process from the input of raw materials to the delivery of the final product. Raw materials are transformed into finished products through a series of operations at stations SA, SB, and SC. Product 1 (P1) goes through operation 1 (O1) at Station A (SA), operation 2 (O2) at Station B (SB), and operation 3 (O3) at Station C (SC); Product 2 (P2) goes through operation 4 (O4) at Station B (SB) and operation 5 (O5) at Station C (SC); and Product 3 (P3) goes through operation 6 (O6) at Station C (SC). These processes also consider setup times, breaks, downtime, and specific operations at each station. This SIPOC helps to clearly visualize how each component of the system is interconnected as a conceptual model of the process.

The case study is focused on a discrete manufacturing system within the metalworking sector, with a process focused on the production of assembly-type products. Where the integration of multiple components in several workstations is required. This type of system is characteristic of industries with high levels of operational complexity, due to the interdependence of processes and the variability in operation and setup times. Although based on an assembly plant in a specific sector, the methodologies employed for its simulation are highly adaptable to other industrial sectors where the assembly process is critical, such as the aerospace, pharmaceutical, and appliance sectors.

### 3.2. Hypothesis

With the initial data collected and the case study clearly defined, the hypotheses that will guide the research are formulated. These hypotheses are focused on assessing the static and dynamic complexity of the manufacturing system. Static complexity refers to the inherent and constant characteristics of the system, while dynamic complexity addresses the changes and fluctuations that occur over time. These hypotheses are established for the purpose of being corroborated or refuted in subsequent stages of the study. To evaluate the variability in complexity in manufacturing systems, the following hypotheses have been put forward:

- Null Hypothesis (HA_0_):
  Changing the production order of the products does not have a significant impact on the static complexity of the workstations.
- Alternative Hypothesis (HA_1_):
  Altering the production order of products significantly impacts the static complexity of workstations.
- Null Hypothesis (HB_0_):
  There is no significant difference in dynamic complexity between workstations.
- Alternative Hypothesis (HB_1_):
  There is a significant difference in dynamic complexity between workstations.

### 3.3. Discrete Event Simulation

According to [51], they define simulation as, “The process of designing and developing a computer model of a real system or process and conducting experiments on this model, with the purpose of understanding the behavior of the system or evaluating various strategies with which the system can be operated”. Later [52], they consider it as, “The development of a mathematical logic model of a system in such a way that it mimics the operation of a real-life process or system over time. Simulation involves the generation of an artificial history of a system, the observation of this history through experimental manipulation, helps us to infer the operational characteristics of such a system”.

In this research for the development of the simulation, we start from a series of techniques such as (i) Monte Carlo simulation, which allows to study the behavior of a system from the generation of random variables, pseudo random numbers, and probability distributions, and (ii) Computer support or simulation programs, which allow to know in depth the operation of a production system or proposed scenarios. The program used is ProModel 10.0, since it allows simulating any type of manufacturing systems, works under Windows with an intuitive graphical interface and object-oriented modeling constructions.

### 3.4. Entropic Complexity Measurement

Shannon entropy has established itself as a fundamental tool for measuring complexity in manufacturing systems due to its ability to quantify uncertainty and diversity in these environments, allowing an accurate assessment of operational complexity in production [53,54]. Its mathematical simplicity and flexibility make it a metric adaptable to different scales and contexts, spanning from the micromanagement of individual processes to the assessment of entire systems [55,56]. Furthermore, Shannon entropy is effective in capturing both static and dynamic complexity, providing a comprehensive view of how this complexity evolves over time [57]. This capability is crucial for identifying areas of optimization within manufacturing systems, where excessive complexity can lead to operational inefficiencies [58,59]. Likewise, Shannon entropy can be integrated with other complexity metrics, such as fractality and network theory, allowing a more complete and deeper analysis of complexity in these systems, reinforcing its role in continuous improvement and innovation in manufacturing [60,61]. Table 3 defines all the variables used in the static and dynamic complexity calculations, ensuring a better understanding and interpretation of the results.

The Shannon entropy *H*(*X*) for a random variable *x* with *n* possible states, where each state *x_i_* occurs with probability *p*(*x_i_*), is defined as:

(1) $$H\left(X\right)=-\overset{n}{\underset{i=1}{\sum }}p(x_{i})log_{b}p\left(x_{i}\right)$$

where:

*p*(*x_i_*) is the probability of the state *x_i_*

log*_b_* is the logarithm in the base *b*. In the case of *b* = 2, the entropy is measured in bits [55].

In manufacturing systems, the states *x_i_* could represent production configurations, machine operating states, or any other relevant variable describing the system.

#### 3.4.1. Measurement of Static Complexity

Consider a manufacturing system with *M* resources. Each resource *i* can be in one of *N* possible states *S*_1_, *S*_2_, *…*, *S_N_*. The probability that the resource *i* is in state *j* is denoted as *P_ij_*. For each resource *i*, the sum of the probabilities over all states must equal 1 [55]. (See Equation (2)):

(2) $$\overset{N}{\underset{j=1}{\sum }}P_{ij}=1\;\;\;\;\;\;\forall \;i=1,2,\ldots ,M$$

The entropy associated with the probability distribution of the states of a resource *i* is denoted as [55]:

(3) $$H_{i}=-\overset{N}{\underset{j=1}{\sum }}P_{ij}log_{2}P_{ij}$$

This measures the uncertainty in the states of the resource *i*. To obtain the total complexity of the system, the entropy is summed over all resources *i*. Equation (4) reflects the static complexity of the system as a whole, considering all possible combinations of resource states at a given time [40,46,62].

(4) $$C_{static}\left(Cs\right)=\overset{M}{\underset{i=1}{\sum }}H_{i}=-\overset{M}{\underset{i=1}{\sum }}\overset{N}{\underset{j=1}{\sum }}P_{ij}log_{2}P_{ij}$$

#### 3.4.2. Measurement of Dynamic Complexity

Dynamic complexity (Cd) refers to how the distribution of system states changes with time. Its equation is:

(5) $$C_{dynamic}\left(Cd\right)=\overset{M}{\underset{i=1}{\sum }}H_{i}\left(t^{’}\right)=-\overset{M}{\underset{i=1}{\sum }}\overset{N}{\underset{j=1}{\sum }}P{’}_{ij}log_{2}P{’}_{ij}$$

where:

*P*’*_ij_* is the probability of state *j* for resource *i* at a later time instant or in a dynamic situation [40,46,62].

### 3.5. Entropy Analysis

For the analysis of results, detailed visualizations are generated, such as heat maps, which illustrate the joint and conditional entropy relationships between the workstations. In addition, a line graph is created that shows the dynamic complexity over a time horizon and by station, allowing patterns and trends in system behavior to be identified. These visualizations are critical to understanding how complexity is distributed within the system and how it varies over time.

### 3.6. Statistical Analysis

In this stage, a statistical analysis is developed based on a sensitivity analysis to evaluate how variations in production sequences and product combinations affect the results obtained, and techniques such as ANOVA are used to corroborate the hypotheses raised about the static and dynamic complexity. This analysis allows us to determine if there are significant differences in complexity between the different workstations. This procedure is crucial to validate the robustness of the findings and ensure that the conclusions are applicable in a variety of operational scenarios. Statgraphic Centurion 19 software is used for its development.

## 4. Results

This section presents the results obtained separated by sections, (i) Discrete event simulation, (ii) Measurement of entropic complexity, (iii) Entropy analysis, and (iv) Statistical analysis.

### 4.1. Discrete Event Simulation Model

**Figure 2:**
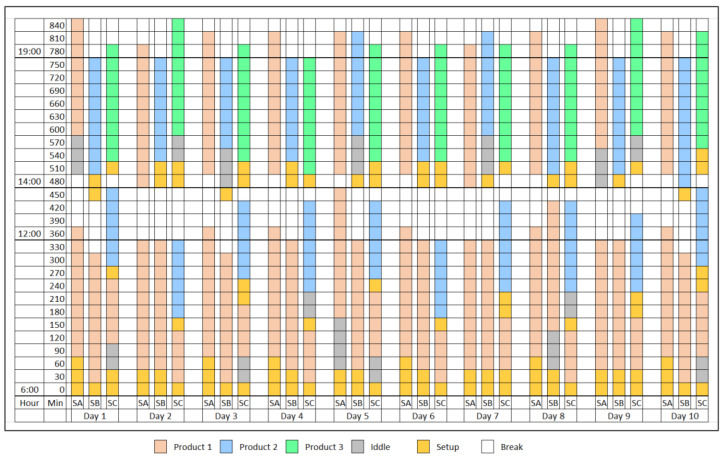
Time distribution per day in manufacturing system for stations.

Discrete event simulation allows decomposing the system into a sequence of individual events occurring at specific points in time, facilitating the detailed representation of complex processes. For its development, Monte Carlo simulation methods are applied to incorporate randomness in the events and generate probability distributions on the results, obtaining a probabilistic view of the key metrics of the system performance and, additionally, a ProModel 10.0 platform is used to build a computational model, allowing experimentation and analysis of different configurations and operational strategies. According to what is described in Figure 2 and taking as input information the scheduling established in each workstation. Considering as assumptions that the system has a start time at six (6:00) hours, then a pause at 12 (12:00) hours, resuming activities at 14 (14:00) hours and ending its production cycle at 19 (19:00) hours and taking into account a sample of 10 (10) working days. Given the above, the simulation from real data is carried out following a structured methodology that integrates the measured data of the real system, considering variables of operation time, readiness time, and rest times. Table 4 shows the Monte Carlo simulation used for workstation A.

Figure 2 presents the simulation of a manufacturing system operating for 10 days, organized in three stations: SA, SB, and SC. Each station manages production depending on the type of product; the system is observed systematically at 30 min intervals, from 6:00 to 19:00. The SA station focuses mainly on the production of Product 1 (P1), while the SB station alternates between the production of Product 1 (P1) and Product 2 (P2). The SC station processes all three products (P1, P2, and P3). In addition to the production periods, the figure details idle times (Idle), machine configurations (Setup), and breaks (Break), which provides a complete view of the daily operation. The colors allow differentiating each product, highlighting regular patterns in production, such as the consistency of SA in manufacturing Product 1 and the operational diversity in SC.

Another modality to be used is by means of a computer package, the program used is ProModel 10.0. The first step in the construction of the model was to enter the required information in the modules used: Locations, Entities, Processing, Arrivals, Attributes, Variables, General Information, and Background Graphics [52].

For modeling purposes, the simulation with termination (where the model is run for a specific period of time, where is the specific event that ends the simulation, therefore the length of the simulation is defined and finite) was taken into account, considering the programming established in each workstation and the assumptions (see items 4.1). Since random variables, such as operating or setup times, are involved, a data analysis was performed using goodness of fit tests to determine the probability distribution associated with the data testing, using the Stat:Fit statistical application. Table 5 shows the results obtained from the ProModel 10.0 by means of the output viewer.

### 4.2. Measurement of Entropic Complexity

According to what is described in Table 1 and taking as input information the programming established in each workstation. Considering as assumptions that the system has a start time at six (6:00) hours, then a break at 12 (12:00) hours, resuming activities at 14 (14:00) hours and ending its production cycle at 19 (19:00) hours. From Equation (4), the necessary calculations are made, considering the observed frequency (Fo), probability (Pr), and entropy (E) of each element. At the end, the total static complexity of each workstation is calculated, highlighting that station C has the highest static complexity with 2.2084 bits, followed by station B with 1.8349 bits, and finally station A as the least structurally complex with 0.9913 bits (see Table 6).

From Equation (5), and from the 26 (26) points of the observation time, with opening time at six (6:00) hours and cutoff time at 19 (19:00) hours, the necessary calculations are made, considering the observed frequency (Fo), probability (Pr), and entropy (E) of each element. Given the above, the total dynamic complexity of each workstation is calculated, highlighting that “station C” has the highest dynamic complexity with 2.396 bits (see Table 7), followed by “station B” with 2.024 bits (see Table 8), and finally “station A” as the one with the lowest dynamic complexity with 1.250 bits (see Table 9).

For the calculation of the dynamic complexity from the results obtained from the simulation with ProModel 10.0 and applying Equation (5), it is highlighted that station C is the most dynamically complex with 1.3184 bits, followed by station B with 1.1069 bits, and finally station A as the least dynamically complex with 0.9436 bits (see Table 10). The same decision compared with that obtained in the Monte Carlo simulation.

The combination of Monte Carlo simulation and ProModel 10.0 in this research is justified by the complementarity of both approaches to perform a comprehensive analysis of static and dynamic complexity in a manufacturing system. Monte Carlo was essential for modeling uncertainty and variability in operation, setup, and downtime, providing a probabilistic view of how these fluctuations impact system complexity. This allowed identifying that Station C is the most complex in both static (2.2084 bits) and dynamic (2.396 bits) terms, due to its operational diversity, while Station A presented the lowest static (0.9913 bits) and dynamic (1.250 bits) complexity. However, Monte Carlo does not allow for detailed real-time workflow analysis. Therefore, ProModel 10.0 was necessary to simulate and visualize the optimization of the operations under different configurations. Through ProModel, the dynamic complexity was evaluated, again highlighting Station C as the most complex (1.3184 bits).

### 4.3. Entropy Analysis Results

**Figure 3:**
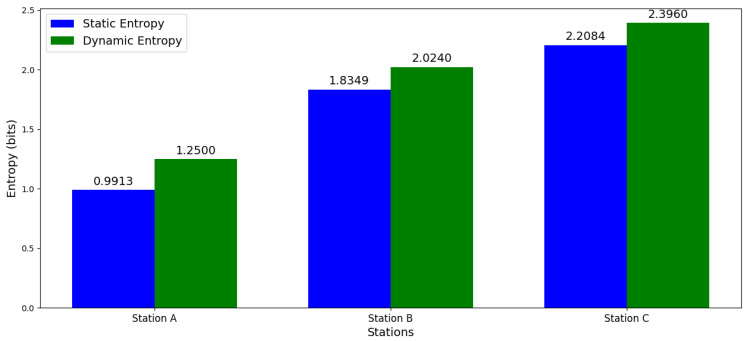
Static vs. dynamic entropy for each station.

Figure 3 compares the static and dynamic entropy for each station, where Station A shows a noticeable increase in dynamic entropy compared to static, indicating that the temporal variability at Station A adds complexity to the system. Station B and Station C also have higher dynamic than static entropy, suggesting that the temporal dynamics at these stations are more complex than the static structure.

**Figure 4:**
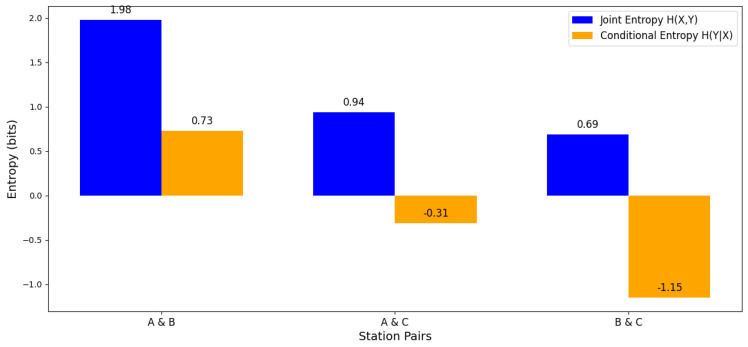
Comparative entropy analysis between stations.

A more in-depth entropic analysis is through the joint entropy between the different workstations. Regarding stations A & B, the joint entropy is 1.978 bits, indicating that the amount of uncertainty or complexity shared between these stations is relatively high. This could suggest a high interdependence or joint operation that generates higher complexity in the system. Stations A & C, with a joint entropy of 0.937 bits, the shared complexity is lower compared to stations A & B. This suggests that stations A & C have less interdependence, or their joint operation is less complex. And stations B & C, the joint entropy is the lowest at 0.686 bits. This could indicate less complexity or interdependence between stations B & C (see Figure 4).

A conditional entropy analysis between the stations identifies that at stations A and B, the conditional entropy is 0.730 bits, which means that, given the knowledge of station A, there is still some uncertainty about the performance of station B. This suggests that the operation of B depends in part on that of A, but not entirely. With respect to stations A and C and B and C, negative values of conditional entropy are evident, this result could indicate that, in the simulation or in the calculation, there are complex or atypical relationships that do not follow the expected behavior. In practice, negative values may be indicative of nonlinear dependencies that the standard conditional entropy formula does not adequately capture (see Figure 4).

**Figure 5:**
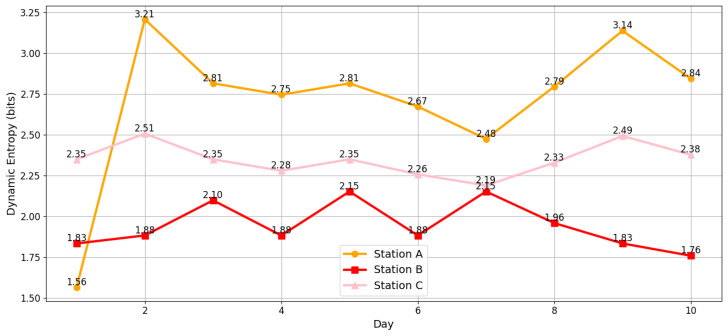
Daily dynamic entropy for each station.

An analysis of the dynamic complexity plot by day and station shows the daily dynamic entropy for three stations (A, B, and C) over 10 days. Figure 5 shows that the dynamic complexity at Station A has significant fluctuations throughout the 10 days, with a maximum peak on day 2, where the complexity reaches a value of 3.21 bits, indicating a high uncertainty in the station’s operations during this day. In addition to this, a great variability in complexity is observed throughout the days, with values ranging from 1.56 bits (Day 1) to 3.21 bits (Day 2). This variability suggests that Station A may be facing significant operational changes or workflow instability issues.

Regarding Station B, the dynamic complexity is more stable compared to Station A, but still shows some notable fluctuations, with peaks on Days 3 and 7 (2.10 and 2.15 bits, respectively) and valleys on Days 1 and 10 (1.83 and 1.76 bits, respectively). These peaks could be associated with specific events that increase uncertainty, such as changes in demand or system configuration. Finally, station C shows the lowest variability in its dynamic complexity, which could indicate a more stable and predictable system, where the entropy remains between 2.26 and 2.49 bits throughout the period, with the highest value recorded on Day 10 (2.49 bits) and the lowest on Day 6 (2.26 bits). This suggests that Station C has relatively consistent behavior, with few interruptions or variations in its workflow.

In synthesis, the analysis suggests that, while Station C operates efficiently, Stations A and B may require improvements in the planning and control of their processes to reduce complexity and improve operational efficiency.

**Figure 6:**
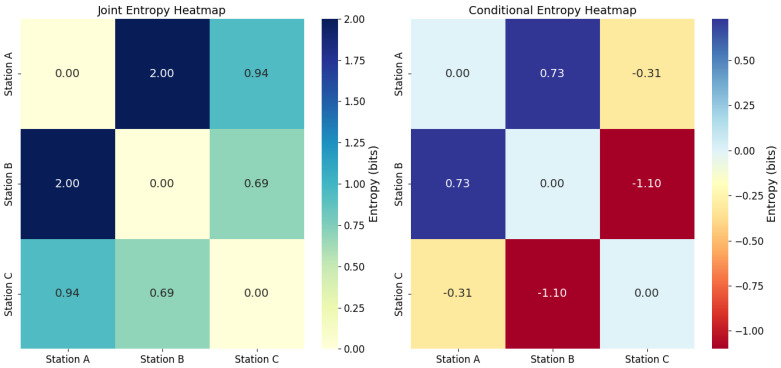
Conjunctive and conditional entropy heat map.

Finally, another important analysis is performed by means of the heat map graph showing the joint and conditional entropy relationships between stations A, B, and C. Figure 6 visualizes how these relationships are distributed, with respect to (i) Joint Entropy Heatmap, which shows how stations A, B, and C are related in terms of joint entropy. Similarly, how these relationships are distributed with respect to (i) Joint Entropy Heatmap, which shows how stations A, B, and C are related in terms of joint entropy; (ii) Conditional Entropy Heatmap, which shows the dependence of one station on another. The analysis reveals that stations B and C are highly correlated, showing the lowest joint entropy (0.69 bits) and the highest mutual dependence with negative conditional entropies (−1.10 bits). This indicates that the state of one significantly reduces the uncertainty about the other. In contrast, stations A and B show the highest joint entropy (2.00 bits). In synthesis, B and C share a strong relationship, while A shows greater independence with respect to the other stations.

### 4.4. Statistical Analysis Results

A sensitivity analysis performed on different production sequences and product combinations has revealed important implications for the management of static complexity at workstations. In the baseline scenario, where the production sequence follows the order P1-P2-P3, the total system complexity is distributed as follows: Station A with 0.9913 bits, Station B with 1.8349 bits, and Station C with 2.2084 bits, accumulating a total of 5.0346 bits. This scenario establishes a benchmark for evaluating how changes in sequencing and product combination affect static complexity.

When analyzing the scenario in which only product P1 is produced, the total system complexity is significantly reduced to 3.2331 bits. Station B shows a reduction in complexity to 1.2302 bits (a 32.96% decrease compared to the base scenario), while Station C also experiences a notable decrease, dropping to 1.0116 bits (54.2% less compared to the base scenario). This result corroborates the hypothesis that simplified production (in this case, focused on a single product) decreases the static complexity of the system.

In the scenario where only P2 is produced, the total complexity is even lower, at 2.6258 bits. Here, Station A reduces its complexity to 0.4154 bits, Station B to 1.1260 bits (a 38.61% decrease from the baseline scenario), and Station C to 1.0844 bits (a 50.9% reduction). This result reinforces the hypothesis that fewer operations distributed among stations leads to lower structural complexity.

**Figure 7:**
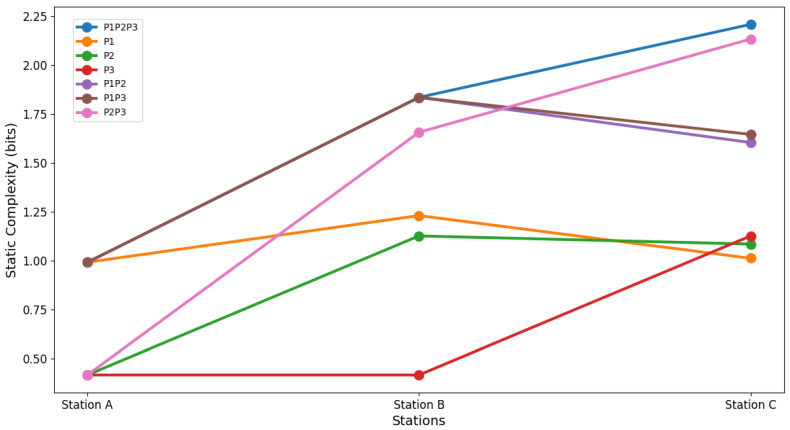
Sensitivity analysis of static complexity with different sequences.

In contrast, when the P1P2 products are produced, the total complexity increases to 4.4299 bits, indicating that the combination of operations of both products increases the complexity at the stations involved, especially at Station C, which, although it reduces its complexity to 1.6037 bits, is still a considerable burden (see Figure 7).

The scenario with the lowest total complexity is the P3 only production, with only 1.9568 bits in total, highlighting a significant reduction in all stations, especially in Station A and B, where complexity remains at 0.4154 bits each. This result suggests that specialization on a single product, particularly one that involves fewer operations, can be an effective strategy to reduce static complexity.

Finally, the results corroborate the hypothesis that the sequence and combination of products have a direct impact on the static complexity of a production system. The null hypothesis, which states that there is no difference in static complexity by altering the sequence or combination of products, can be rejected based on the data obtained. Instead, the alternative hypothesis, which suggests that changes in the sequence and combination of products affect static complexity, particularly in stations with multiple operations such as stations B and C, is accepted. This analysis provides a solid basis for optimizing production planning to minimize operational complexity and improve system efficiency.

Using ANOVA analysis, where the static complexity variance is decomposed into two components: a between groups component and a within groups component. The F-ratio, which in this case is equal to 6.23197, being the ratio between the between group estimate and the within group estimate. Since the *p*-value of the F test is less than 0.05, it is determined that there is a statistically significant difference between the mean Static Complexity, between one station and another, with a confidence level of 95.0%.

**Figure 8:**
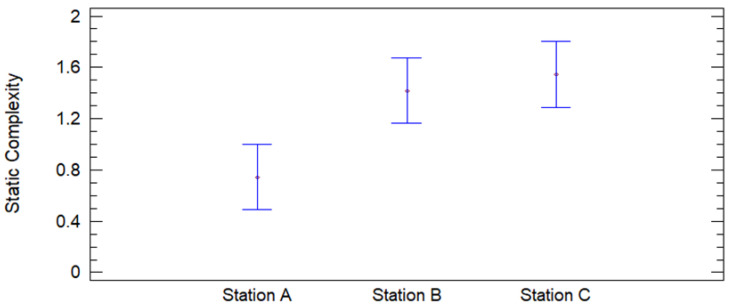
Graph of means for static complexity.

The graph of means shows the result of a statistical analysis comparing the static complexity between stations A, B, and C. The graph includes 95% confidence intervals for each of the stations, allowing an assessment of the statistical significance of the observed differences in the static complexity means. This analysis uses Fisher’s LSD (Least Significant Difference) method to determine if the differences between means are statistically significant. From Figure 8 it is evident that (i) The station A static complexity mean is the lowest of the three, around 0.8 bits. This aligns with what was observed in the previous analysis, where Station A showed the lowest static complexity and lowest variability; (ii) station B’s mean is around 1.4 bits, indicating a higher static complexity compared to station A, this station shows a significant increase in static complexity; and (iii) Station C has a mean static complexity close to 1.6 bits, being the highest among the three stations. However, the difference between the means of Station B and Station C does not appear to be very large. Since the confidence interval of station A does not overlap with those of stations B and C, the null hypothesis (HA_0_) can be rejected for the comparison between station A and the other two stations. However, the null hypothesis (HA_0_) cannot be rejected for the comparison between station B and station C, suggesting that their means are not significantly different in this analysis. Given the above, the alternative hypothesis (HA1), which states that there are significant differences in the mean static complexity, is accepted.

**Figure 9:**
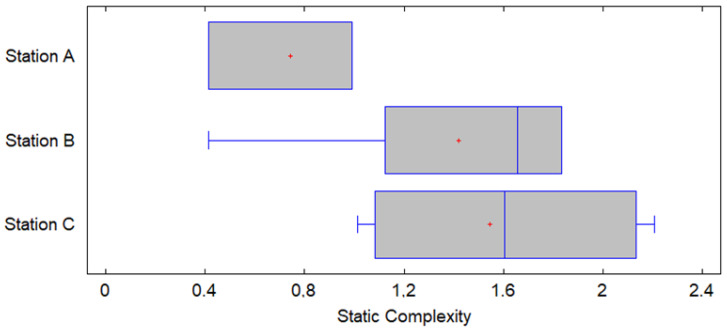
Graph box plot of static complexity.

The hypothesis is also corroborated by the box plot, which suggests that the null hypothesis (HA_0_) could be rejected, given that there are evident differences in the medians and ranges of complexity between stations, especially between station A and station C (see Figure 9).

Regarding the analysis of dynamic complexity, the ANOVA from the F ratio, determines that it is equal to 18.6138, with a *p*-value of the F test less than 0.05, where it is determined that there is a statistically significant difference between the mean of Dynamic Complexity between workstations with a level of 95.0% confidence. Given the above, it can be concluded with confidence that the dynamic complexity varies between stations, and that these differences are not due to chance. This suggests that the behavior in terms of dynamic complexity is different for each station in the manufacturing process.

**Figure 10:**
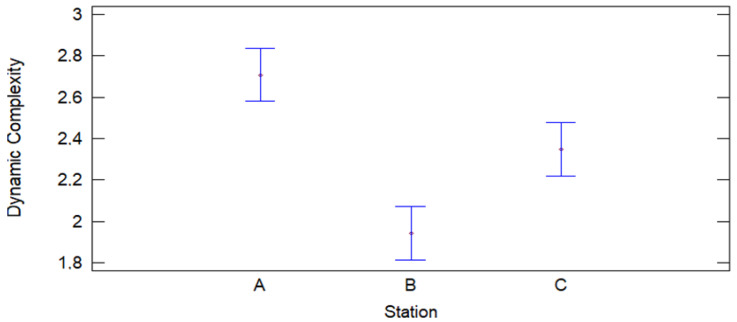
Graph of means for dynamic complexity.

The plot of means with Fisher LSD 95% confidence intervals for dynamic complexity at Stations A, B, and C shows statistically significant differences between station means. Station A has the highest mean dynamic complexity, followed by Station C, while Station B has the lowest mean. The confidence intervals do not overlap, indicating that the differences in dynamic complexity between stations are significant. This suggests that each station faces different levels of operational complexity, reinforcing the need to adopt customized strategies to optimize efficiency at each station. These results corroborate the hypothesis that dynamic complexity varies significantly between stations (see Figure 10).

**Figure 11:**
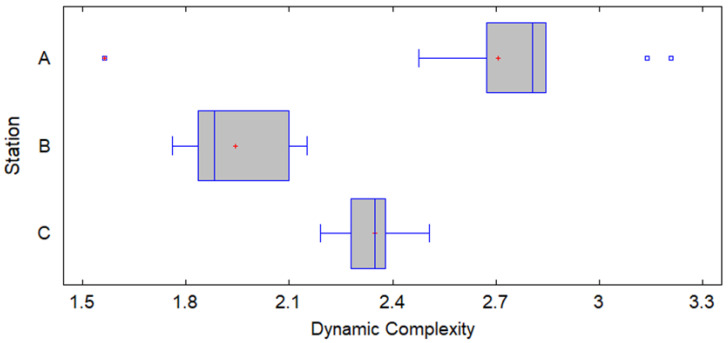
Graph box plot of dynamic complexity.

More broadly, the analysis of the dynamic complexity through the box plot reveals significant differences between stations A, B, and C. Station A shows the highest variability and presence of outliers, suggesting the need to optimize processes and consider the implementation of Industry 4.0 technologies to stabilize its performance. In contrast, Station B, although more stable, could benefit from further standardization of procedures to reduce the slight variability observed. Station C, with the lowest variability and lowest complexity, stands out as a model of efficiency that could serve as a reference for the other stations (see Figure 11). These observations corroborate the alternative hypothesis (HB_₁_), which proposes that there are significant differences in dynamic complexity between stations, corroborated by the previous ANOVA analysis, suggesting a station-specific approach to process optimization.

## 5. Discussion

The research developed in this study has demonstrated the importance of integrating entropic methods with discrete event simulation for the accurate and detailed measurement of complexity in manufacturing systems. The main contributions of this work are the following: (i) Presentation of a methodology for complexity measurement, integrating entropic methods and discrete event simulation techniques, allowing an accurate and detailed assessment of both static and dynamic complexity in manufacturing systems. (ii) Development of an approach based on dynamic simulation for complexity measurement in manufacturing systems, overcoming the limitations of traditional methods that focus mainly on static complexity measurement. (iii) Extensive entropic analysis by means of Shannon’s measurement and use of comparative and conditional measures, an innovative element for the scientific, academic and industrial community, which overcomes the current gaps in research work. (iv) Validation in a practical case, demonstrating its viability and applicability in future research in the industrial field.

The results obtained have corroborated the alternative hypothesis for static complexity, showing that factors such as production sequence significantly influence the structural complexity of workstations. This approach is crucial for optimizing efficiency and minimizing bottlenecks in production systems. For example, the static complexity at Station A ranged from 0.4154 to 0.9913 bits, being consistently lower compared to Stations B and C, which reached up to 2.2084 bits in the most complex scenario. These data highlight the usefulness of the methodology developed to anticipate and manage complexity in a manufacturing environment. Additionally, the stability in the observed operation frequencies suggests that dynamic complexity is not significantly affected by changes in the production sequence, thus corroborating the null hypothesis for dynamic complexity, which states that alterations in the production sequence do not modify the probability or entropy of operations at each station.

The results of this research have both theoretical and practical implications of great relevance to the field of manufacturing. From a theoretical perspective, the development of a comprehensive methodology that combines Shannon information theory with discrete event simulation techniques significantly expands the conceptual framework for measuring complexity in manufacturing systems, offering a new tool to simultaneously analyze static and dynamic complexity. This approach overcomes the limitations of traditional models, which focus on static perspectives, and opens up new lines of research to study the influence of dynamic and structural factors on the efficiency of production systems. On a practical level, the results validate the applicability of this methodology in a real environment of the metal-mechanical sector, providing manufacturing managers with a robust framework to anticipate, measure, and manage complexity. This translates into an optimization of the sequence of operations and the reduction of bottlenecks, which directly contributes to improving the efficiency, stability, and competitiveness of production processes. Furthermore, the versatility of the methodology to be adapted to other industrial sectors, such as aerospace and pharmaceuticals, underlines its usefulness as a strategic tool for operational planning and the design of more resilient and sustainable systems.

These findings are crucial for decision-making in production management, suggesting that an optimization in the sequence of operations and an adjustment in the production volume can contribute significantly to reduce structural complexity, thus improving the overall efficiency of the process. The integration of discrete event simulation in this analysis provides an additional tool for forecasting and mitigating the effects of complexity in manufacturing systems, offering a more robust framework for operational planning and management.

## 6. Conclusions

This research work has developed a detailed analysis of static complexity in manufacturing systems, using an integral methodology that ranges from a case study to a robust statistical analysis, including discrete event simulation and entropy measurement. The proposed methodology consists of six stages: identification of the manufacturing system, formulation of hypotheses, simulation of discrete events, measurement of complexity through Shannon entropy, comparative analysis of entropy and, finally, validation of hypotheses by means of ANOVA. The results obtained in this work have allowed us to corroborate the alternative hypothesis for static complexity, demonstrating that factors such as production sequence and product volume have a significant impact on the structural complexity of the workstations. For example, the static complexity for Station A was consistently lower (0.4154 to 0.9913 bits) compared to Station B and C, which showed higher variations and complexity (up to 2.2084 bits in Station C). These findings are fundamental to understanding how production planning can optimize efficiency and reduce bottlenecks in manufacturing systems. The discrete event simulation-based approach has been shown to be effective in predicting and managing complexity in production systems, providing a quantitative framework that can guide strategic decision-making. In addition, the integration of statistical analyses such as ANOVA has rigorously validated the effects of different variables on system complexity, reinforcing the validity of the results obtained. The findings of this study have significant implications for the management of manufacturing systems by providing a quantitative analytical framework that facilitates strategic decision-making. The proposed methodology allows identifying and mitigating factors that increase structural complexity, thus optimizing production planning and reducing operational inefficiencies. The integration of entropy measures and robust statistical analysis provides a rigorous approach to design management strategies that minimize bottlenecks and increase system resilience to operational variabilities. For future work, it is recommended to expand this analysis to other manufacturing systems with varied operational configurations and to consider the inclusion of additional dynamic factors, such as demand variability and possible interruptions in production. It is also suggested to explore the use of advanced optimization techniques, such as artificial intelligence algorithms, to develop production sequences that minimize structural complexity more efficiently and effectively.

## Figures and Tables

**Table 1:** Main gaps and challenges in the literature.

Authors	Gaps	Authors	Challenges
Klamut et al., 2020 [13]; Chen and Lin 2021 [14]; Herrera-Vidal et al., 2024 [15].	Limited application to real industrial settings	Huo et al., 2020 [16]; Zhang et al., 2021 [17]; Ponce et al., 2020 [18].	Expanding to broader and more diverse systems
Tong et al., 2023 [19]; Ponce et al., 2020 [18]; Xu et al., 2021 [20]; Herrera-Vidal et al., 2024 [15].	Few validations in diverse systems	Klamut et al., 2020 [13]; Tong et al., 2023 [19]; Chen and Lin, 2021 [14].	Empirical validation in complex industrial settings
Martínez-Olvera, 2020 [21]; Vidal et al., 2023 [12]; Zhang et al., 2020 [22].	Mainly theoretical formulations	Fan et al., 2023 [23]	Integrating human machine interaction over time
Fan et al., 2023 [23].	Lack of human-machine interaction considerations	Zhang et al., 2020 [22]; Wang and Jin, 2020 [24].	Applying to other manufacturing contexts
Wang and Jin 2020 [24]; Zhang et al., 2020 [22].	Restricted to specific types of flows or volatility	Wang et al., 2020 [25]; Herrera-Vidal et al., 2024 [15]	Implementing in real time, embedded, or portable systems

**Table 2:** Sipoc of the manufacturing process.

Suppliers	Inputs	Process	Outputs	Customers
Supplier of raw materials	Raw materials (P1, P2, P3)	Receipt	Raw materials (P1, P2, P3)	Station SA, SB, SC
Supplier of machinery	Machine SA, SB, SC		Machinery (P1, P2, P3)	
Supplier of labor	Operator SA, SB, SC		Labor (P1, P2, P3) (P1, P2, P3)	
Supplier of energy	Energy SA, SB, SC		Energy (P1, P2, P3)	
Receipt	Raw materials; machines; operators; energy	SA Station (O1 for P1)	Subassembly (SA for P1)	Station SB
SA station (O1 to P1)	Subassembly (SA for P1)	Station SB (O2 for P1)	Subassembly (SB for P1)	Station SC
Station SB (O2 for P1)	Subassembly (SB for P1)	Station SC (O3 for P1)	Product 1 (P1)	End Customer
Reception	Raw materials; machines; operators; energy	Station SB (O4 for P2)	Subassembly (SB for P2)	Station SC
SB Station (O4 for P2)	Subassembly (SB for P2)	SC Station (O5 for P2)	Product 2 (P2)	End Customer
Reception	Raw materials; machines; operators; energy	SC Station (O6 for P3)	Product 3 (P3)	End Customer

**Table 3:** Notation and description of variables used in complexity measurement.

Notation	Description
H(X)	Shannon entropy for a random variable X, representing uncertainty in bits.
x_i_	Possible states of the system or resource being measured.
P(x_i_)	Probability that the system is in state x_i_
Log_2_	Logarithm base 2, used to measure entropy in bits.
C_static_	Static complexity of the system, measuring uncertainty at a specific point in time.
M	Total number of resources or elements in the system.
N	Number of possible states for each resource.
P_ij_	Probability that resource i is in state j.
H_i_	Entropy of resource i, indicating the uncertainty in its states.
C_dynamic_	Dynamic complexity, analyzing changes in the system’s states over time.
P’_ij_	Probability that resource i is in state j at a future time t′.
H_i_(t’)	Entropy of resource iii at time t′, representing the dynamic uncertainty.
t’	Future point in time used to measure dynamic complexity.

**Table 4:** Monte Carlo simulation for station A.

Event	Start	Setup	State	Operation	Idle	Final Hour	Break	State	Operation	Idle	End Time
Day 1	6:00:00	1:02:30	O1	5:01:30	0:00:00	12:03:59	14:03:59	Idle	5:29:18	1:13:36	20:46:53
Day 2	6:00:00	0:57:58	O1	4:53:40	0:00:00	11:51:37	13:51:37	O1	5:33:43	0:00:00	19:25:21
Day 3	6:00:00	1:14:16	O1	5:03:58	0:00:00	12:18:13	14:18:13	O1	5:18:09	0:00:00	19:36:22
Day 4	6:00:00	1:02:19	O1	5:01:17	0:00:00	12:03:36	14:03:36	O1	5:31:20	0:00:00	19:34:57
Day 5	6:00:00	1:12:57	Idle	5:06:08	1:25:24	13:44:30	14:19:06	O1	5:33:18	0:00:00	19:52:24
Day 6	6:00:00	1:10:44	O1	5:03:15	0:00:00	12:14:00	14:14:00	O1	5:32:59	0:00:00	19:46:59
Day 7	6:00:00	0:49:15	O1	4:58:22	0:00:00	11:47:36	13:47:36	O1	5:29:33	0:00:00	19:17:09
Day 8	6:00:00	1:14:10	O1	5:02:35	0:00:00	12:16:45	14:16:45	O1	5:22:20	0:00:00	19:39:05
Day 9	6:00:00	0:57:11	O1	4:56:46	0:00:00	11:53:58	13:53:58	Idle	5:37:53	1:27:10	20:59:01
Day 10	6:00:00	1:03:01	O1	5:02:37	0:00:00	12:05:38	14:05:38	O1	5:33:47	0:00:00	19:39:25

**Table 5:** ProModel 10.0 simulation results of the case study.

	Operation (%)	Setup (%)	Idle (%)	Wait (%)	Block (%)	Down (%)	Total
Station A	8.81	5.33	3.71	0.00	82.15	0.00	100.00
Station B	15.66	5.07	3.44	0.00	75.83	0.00	100.00
Station C	63.13	18.30	18.57	0.00	0.00	0.00	100.00

**Table 6:** Static complexity measurement results.

	Station A			Station B			Station C		
	Fo	Pr	E	Fo	Pr	E	Fo	Pr	E
Operation 1	20	0.769	0.2912						
Operation 2				10	0.385	0.5302			
Operation 3							4	0.154	0.4155
Operation 4				9	0.346	0.5298			
Operation 5							6	0.231	0.4882
Operation 6							9	0.346	0.5298
Setup	2	0.077	0.2846	3	0.115	0.3595	3	0.115	0.3595
Break	4	0.154	0.4155	4	0.154	0.4155	4	0.154	0.4155
Total	26	1.000	0.9913	26	1.000	1.8349	26	1.000	2.2084

**Table 7:** Results of the dynamic complexity measurement at station C.

	Observations										Station C		
	Day 1	Day 2	Day 3	Day 4	Day 5	Day 6	Day 7	Day 8	Day 9	Day 10	Fo	Pr	E
O3	5	4	4	4	5	4	4	4	4	5	43	0.165	0.4294
O5	6	6	6	7	6	6	7	7	6	6	63	0.242	0.4955
O6	8	6	8	9	8	8	8	8	6	7	76	0.292	0.5187
Setup	4	4	5	3	4	4	5	4	5	5	43	0.165	0.4294
Idle	2	2	2	2	2	0	0	2	2	2	16	0.062	0.2475
Break	1	4	1	1	1	4	2	1	3	1	19	0.073	0.2758
Total	26	26	26	26	26	26	26	26	26	26	260	1.000	2.396

**Table 8:** Results of the dynamic complexity measurement at station B.

	Observations										Station B		
	Day 1	Day 2	Day 3	Day 4	Day 5	Day 6	Day 7	Day 8	Day 9	Day 10	Fo	Pr	E
O2	10	10	10	10	10	10	10	10	10	10	100	0.385	0.530
O4	9	8	7	8	6	8	6	9	9	10	80	0.308	0.5232
Setup	3	4	2	4	3	4	3	3	3	2	31	0.119	0.3658
Idle	0	0	3	0	3	0	3	3	0	0	12	0.046	0.2048
Break	4	4	4	4	4	4	4	1	4	4	37	0.142	0.400
Total	26	26	26	26	26	26	26	26	26	26	260	1.000	2.024

**Table 9:** Results of the dynamic complexity measurement at station A.

	Observations										Station A		
	Day 1	Day 2	Day 3	Day 4	Day 5	Day 6	Day 7	Day 8	Day 9	Day 10	Fo	Pr	E
O1	16	20	19	19	19	19	20	19	17	19	187	0.719	0.342
Setup	3	2	3	3	3	3	2	3	2	3	27	0.104	0.3393
Idle	3	0	0	0	3	0	0	0	3	0	9	0.035	0.168
Break	4	4	4	4	1	4	4	4	4	4	37	0.142	0.4003
Total	26	26	26	26	26	26	26	26	26	26	260	1.000	1.250

**Table 10:** Results of dynamic complexity measurement with ProModel 10.0.

	Operation (%)	Setup (%)	Idle (%)	Wait (%)	Block (%)	Down (%)	Total
Station A	0.3088	0.2254	0.1763		0.2330		0.9436
Station B	0.4189	0.2181	0.1672		0.3027		1.1069
Station C	0.4189	0.4484	0.4511				1.3184
Total							3.3688

## Data Availability

The original contributions presented in the study are included in the article. Further inquiries can be directed to the corresponding authors.
